# Epigenetic Control of Human Endogenous Retrovirus Expression: Focus on Regulation of Long-Terminal Repeats (LTRs)

**DOI:** 10.3390/v9060130

**Published:** 2017-05-31

**Authors:** Tara P. Hurst, Gkikas Magiorkinis

**Affiliations:** 1 Department of Zoology, University of Oxford, Oxford OX1 3PS, UK; tara.hurst@zoo.ox.ac.uk; 2 Department of Hygiene, Epidemiology and Medical Statistics, Medical School, National and Kapodistrian University of Athens, 11527 Athens, Greece

**Keywords:** Endogenous retroviruses, HERVs, LTR, epigenetics, Krüppel-associated box zinc finger protein, KRAB-ZFP

## Abstract

Transposable elements, including endogenous retroviruses (ERVs), comprise almost 45% of the human genome. This could represent a significant pathogenic burden but it is becoming more evident that many of these elements have a positive contribution to make to normal human physiology. In particular, the contributions of human ERVs (HERVs) to gene regulation and the expression of noncoding RNAs has been revealed with the help of new and emerging genomic technologies. HERVs have the common provirus structure of coding open reading frames (ORFs) flanked by two long-terminal repeats (LTRs). However, over the course of evolution and as a consequence of host defence mechanisms, most of the sequences contain INDELs, mutations or have been reduced to single LTRs by recombination. These INDELs and mutations reduce HERV activity. However, there is a trade-off for the host cells in that HERVs can provide beneficial sources of genetic variation but with this benefit comes the risk of pathogenic activity and spread within the genome. For example, the LTRs are of critical importance as they contain promoter sequences and can regulate not only HERV expression but that of human genes. This is true even when the LTRs are located in intergenic regions or are in antisense orientation to the rest of the gene. Uncontrolled, this promoter activity could disrupt normal gene expression or transcript processing (e.g., splicing). Thus, control of HERVs and particularly their LTRs is essential for the cell to manage these elements and this control is achieved at multiple levels, including epigenetic regulations that permit HERV expression in the germline but silence it in most somatic tissues. We will discuss some of the common epigenetic mechanisms and how they affect HERV expression, providing detailed discussions of HERVs in stem cell, placenta and cancer biology.

## 1. Introduction

The human genome is littered with endogenous retroelements, including non-long-terminal repeat (LTR) elements such as long interspersed nuclear repeats (LINEs) and short interspersed nuclear repeats (SINEs), as well as the long-terminal repeats (LTR)-containing endogenous retroviruses (ERVs). It is widely accepted that retroelements are subject to repression by both genetic and epigenetic mechanisms. However, much of what is known has been elucidated through studies in other species, such as mice. While informative, such work is limited by differences between the species, particularly differences in ERV activity. For example, ERVs in mice are much more active than in humans and produce infectious particles [1] which have not yet been demonstrated in humans. Indeed, there seems to be a negative correlation between ERV proliferation and body size, suggesting that ERV numbers scale with the number of cells of the host so a mouse is more likely to have active ERVs relative to humans based on body size. Further, tumorigenic insertional mutagenesis is more likely to happen when more cells have ERV proliferation [2]. It seems that a complete knock-out of the ERVs (e.g., through deleterious mutations) would be the safest option for the host but this would lead to a complete extinction of ERVs. Intuitively, the “sweet spot” of ERV activity that allows both ERVs and host survival could be a window of activity near early life stages (e.g., germline and embryonic stem cells) where the number of cells is irrelevant to the final body size of the host, followed by silencing in somatic tissues. This arrangement is likely to be served through epigenetic silencing of ERVs. Here, we will describe some of the recent findings on the regulation of ERVs by epigenetics, emphasising studies on human ERVs (HERVs) in normal tissues and in diseases.

## 2. Human Endogenous Retroviruses

HERVs were detected in human cells in the 1970s, with early descriptions of retrovirus-like particles in placentae [3] and germ line cancers [4]. The Human Genome Project revealed that HERVs comprise 8% of the genome [5]. HERVs ERVs belong to a number of distinct families that integrated independently during evolution [6]. One of these families, HERV-K HML-2 (HK2), emerged prior to the divergence of hominids from Old World monkeys. However, a number of human-specific integrations have been identified [7]. Further, there is evidence of recent activity of HK2 within the human genome, such as the discovery of polymorphic integrations of HK2 [8,9], including an intact provirus located on the X chromosome [10]. The vast majority of HERVs are rendered inactive by an accumulation of mutations, as well as by epigenetic mechanisms. Despite this, there are numerous reports implicating HERV expression, particularly of HK2, in autoimmune diseases and cancer [11,12,13,14]. Thus, the presence, activity and expression of HERVs is of great interest. 

Control of HERV expression depends upon regulation at the level of the LTRs. These function as promoters for HERV expression [15], have strong RNA Polymerase II regulatory sequences [16,17], and contain a plethora of transcription factor binding sites [18]. The LTRs can bind nuclear transcription factors [19] and more recently have been shown to be responsive to pro-inflammatory cytokines in a cellular model of amyotrophic lateral sclerosis (ALS) [20]. Importantly, solo LTRs are present in the genome due to recombination that excises the rest of the provirus [21]. Indeed, up to 85% of HERVs have undergone this recombinatorial deletion [22], making most HERV loci solo LTRs. Solo LTRs can serve as promoters in both sense and antisense orientations [23] and can alter the expression of host genes [24,25]. Further, the expression of very long intergenic RNAs (vlincRNAs) which control pluripotency and malignancy was HERV LTR-driven [26], suggesting a role for HERV LTRs in regulating not only protein-coding genes but also the expression of long non-coding RNAs. Thus, the LTRs are an important site for epigenetic modifications to control HERV and human gene expression. 

## 3. Epigenetics

Epigenetic regulation includes the modification of both DNA and the histones around which DNA is wound to create chromatin [27]. The formation and packing of the chromatin is itself an epigenetic mechanism; tightly-packed chromatin is associated with gene silencing and vice versa. Regulation is also achieved by the modification of nucleotides and proteins by the addition of chemical groups, such as methyl or acetyl groups. For example, modification of the histone H3 by trimethylation of lysine 4 (H3K4me3) is associated with gene activity, while that of lysine 9 (H3K9me2/3) or 27 (H3K27me3) defines condensed chromatin packing and gene silencing [27]. There is also a strong association between DNA methylation and the H3K9me3 mark [28].

One way to understand epigenetic system is by considering it to comprise writers, readers and erasers of the modifications; these are enzymes that add, bind to or remove chemical groups e.g., methyl groups from DNA [29]. Strategies to study epigenetics include the use of drugs which inhibit DNA or histone modifying enzymes (the ‘writers’ or ‘erasers’). For example, the nucleoside analogue 5′-azacytidine (5′-aza) is incorporated into cellular DNA and inhibits the DNA methyltransferase 1 (DNMT1), resulting in passive demethylation and reactivation of silenced genes [30]. Similarly, the use of histone deactylase inhibitors (HDACi) results in the retention of acetyl groups on the histones and therefore of active gene expression [31]. We will first describe current epigenetic mechanisms of control of HERVs and then discuss specific examples in more detail.

## 4. Epigenetic Regulation of Human Endogenous Retroviruses

The studies that have been done on HERVs suggest that multiple control strategies are used: localisation of proviruses to heterochromatin, chromatin packing to block access to the LTRs, CpG methylation and histone deacetylation contribute to the control of HERVs in the genome. The predominant view is that these epigenetic mechanisms keep HERVs silenced [32,33]. However, it is also possible that a more nuanced view allows epigenetics a role in transcriptional regulation rather than silencing alone. This idea is suggested by transcriptome studies which report that up to one-third of all HERV loci are transcribed [34], a number that would not make sense if the epigenetic repression were not somewhat ‘leaky’. 

### 4.1. CpG Methylation

It is usual for CpG nucleotides to be methylated throughout the human genome, including those found in HERVs; exceptions to this, referred to as CpG islands (CGIs), are sites of low methylation that are frequently found near active genes and enhancer elements [35]. The methylation of CpGs is carried out by DNMTs, with DNMT1 being the maintenance methyltransferase which is important for fidelity of methylation during DNA replication [35]. A microarray study analysing HERV families throughout the genome found that HERVs are heavily methylated in normal tissues [36]. Further, the age of the HERVs correlates with their methylation status, with a loss of methylation appearing in older families such as HERV-H [36]. CpG methylation is a critical mechanism of silencing and has been demonstrated for the HK2 5′ LTR in germ cell tumours (GCTs) [37]. In this study, methylation of 5′ LTRs correlated with transcriptional suppression in the Tera-1 cell line [37]. Importantly, the effect is cell-type dependent, implying that other factors are also critical for regulation of HERV expression. These can include transcription factors, as well as other types of epigenetic modifications. 

### 4.2. Histone Acetylation

Acetylation of lysine residues in histones is catalysed by histone acetyltransferases (HATs) and removed by histone deacetylases (HDACs) [38]. Histone acetylation blocks the positive charges on lysine residues which destabilises chromatin and favours transcriptional activation; deacetylation stabilises chromatin and thus leads to transcriptional repression [38]. This has led to the use of HDAC inhibitors (HDACi) to reverse HIV-1 latency as part of a ‘kick and kill’ approach to curing HIV-1 infection [39]. We were interested in whether the transcriptional activation resulting from HDACi treatment of HIV-1-infected cells would also activate HERVs [40]. To test this, we examined the expression of particular HERV families (HK2, HERV-W, HERV-FRD) following HDACi treatment using quantitative RT-PCR with Molecular Beacons probes. Indeed, we found that HDACi treatment did not significantly up-regulate the HERVs in either latency cell lines or primary T cells infected with HIV-1 [40]. This implies that histone deacetylation alone is not responsible for HERV repression, a finding consistent with the importance of other factors, particularly CpG methylation, in silencing HERVs. For example, the combination of the HDACi trichostatin A (TSA) and 5′-aza increased HERV-Fc1 expression in HEK 293s, whereas TSA alone did not [30]. However, the same study did find that TSA alone or in combination with 5-aza increased in HERV-Fc1 expression in peripheral blood mononuclear cells (PBMCs) [30]. The different results could be due to the distinct cell types used, with cancer cell lines being expected to differ from PBMCs.

### 4.3. Histone Methylation, Heterochromatin and Krüppel-associated box zinc finger proteins (KRAB-ZFP)

The differential methylation of histones is critical to the activation or repression of genes. In particular, methylation of histone H3 at different lysine residues is an indicator of activity; the predominant marks are H3K9me3 (activity) and H3K4me3 (silencing). In a bioinformatics study, HERV-K was found predominantly in areas of repressed chromatin and there was a strong association with H3K9me3 [41]. In comparison, the localisation of genomic HERVs was in sites of inactive chromatin (older HERV proviruses) or an intermediate position (younger HERVs) [42]. This may be evidence of purifying selection [42], with HERVs that are found in active genetic regions being selected against over time and HERVs found within heterochromatin being retained. The reconstituted HERV (HERV-Kcon) was found to preferentially integrate near active chromatin marks including H3K4me1 and 2 as well as CpG islands [42]. HERV-Kcon is a lab reconstruction of a potential progenitor of HERV-K (HML-2), derived from a consensus sequence. By being integrated near active chromatin marks, it is behaving as a ‘young’ virus.

One critical system that contributes to histone methylation and heterochromatin formation early in the embryo are Krüppel associated box zinc finger proteins (KRAB-ZFP). The zinc finger domain binds to DNA in a sequence-specific manner, allowing the recruitment of other proteins via the KRAB domain; in particular, the scaffold protein TRIM28/KAP1, forming part of a larger protein complex that modifies the histones [43]. These proteins include DNMT1, and DNMT3a/b, as well as the histone lysine methyltransferase, SETDB1, which is responsible for the H3K9me3 modification [28]. The majority of human KRAB-ZFP binding sites were located within transposons, mainly retrotransposons including HERVs [44]. The KRAB-ZFP bind to HERVs and silence them by burying them in heterochromatin. 

Indeed, the LTR-containing retrotransposons have been found to co-evolve with KRAB-ZFP genes [43]. The authors propose a model in which the threat to the genome of each new integration leads to the emergence of new KRAB-ZFP genes [43]. This was supported by a later study showing that the integration of each family of HERVs coincided with a new KRAB-ZFP [45]. This occurs via positive selection of divergent KRAB-ZFP genes, particularly in the region coding for DNA contact residues in the protein [43]. Thus, evolution of KRAB-ZFP genes allows the protein to bind to novel DNA sequences found in newly-integrated retroelements. More recently, it was found that the KRAB-ZFPs that recognise HERVs and LINEs emerged in the same last common ancestor as their target retrotransposons [44], providing further support for the co-evolution hypothesis. 

In addition, it has been suggested that genomic imprinting emerged from the use of epigenetics to deal with the retroviral burden and this is supported by the existence of KRAB-ZFP genes involved in imprinting [43]. For example, Zfp57 recruits TRIM28/KAP1 complexes to imprinted differentially methylated regions (DMRs) and maintains methylation during the pre-implantation period in the embryo [28]. Zfp57 is highly expressed in embryonic stem cells but then down-regulated in adult tissues except the ovaries and testes [28]. Zfp57 is also predicted to bind to motifs present in the HERVS71-int family [45]. One hypothesis is that this initial suppression by DNA methylation in embryonic development obviates the need for further involvement of the KRAB-ZFP in HERV silencing in adult tissues. However, not all HERVs may be silenced during imprinting. For example, not all LTRs in mice are suppressed by KRAB-ZFP in oocytes and during embryonic development [46] and this is thought to possibly allow transcripts of ERVs or chimeric ERV/host gene transcripts to persist and assist in development [47]. One possibility is that this escape from repression permits the novel use of the LTRs as alternative promoters of genes [46]. Whether this occurs in human oocytes and embryos is not clear; the ethics of studying human embryos also makes this difficult to determine and hence we have included mouse studies here.

Likewise, the function of another effector protein recruited to KRAB-ZFP complexes, SETDB1, has been studied more intensively in mice, where it was found to be critical for global repression of ERVs [48]. The loss of SETDB1 resulted in up-regulation of ERVs but this depended upon the presence of the particular transcription factors; there was a ‘functional match’ between transcription factor expression and the ERV LTRs [48]. Further, the de-repressed ERVs could have a causal effect on altered gene expression of proximal genes [48]. This study showed that SETDB1 was responsible for histone methylation and ERV repression in lineage-committed adult cells, in this case B cells [48]. Similarly, KRAB-ZFPs and KAP1 were found to control transposable elements in adult tissues [49]. Thus, there is a role for the KRAB-ZFPs beyond imprinting in the ongoing epigenetic regulation of ERVs. More research into the function of these proteins in human tissues is needed to fully understand the regulation of HERVs.

### 4.4. Nucleosomal Positioning

There is a growing appreciation of the role of nucleosome position in the regulation of gene expression [50]. The regulation of HERV expression by nucleosomal positioning was postulated almost 20 years ago [51]. The HERV-K LTR was found to lack the TATA box promoter and to not use an initiator sequence in its place; thus, initiation of transcription was by a distinct mechanism involving the cellular transcription factors Sp1 and Sp3 [52]. The HERV-K LTR contains multiple transcription start sites (TSS), with one of these forming the major TSS and the others being dispersed sites [52]. The authors hypothesised that the Sp1 and Sp3 binding to the LTR freed the TSS from nucleosomes, allowing transcription [52]. Recently, the use of alternative transcription start sites (TSS) was described as a positive mechanism to regulate LTR-directed transcription [53]. Critical to this was altered nucleosomal occupancy; in normal cells, this functions to keep retrotransposons silenced by the production of truncated transcripts [53]. In contrast, reduced nucleosomal occupancy in stressed cells altered the usage of TSS to permit full-length transcripts [53]. Thus, while nucleosomal positioning can suppress HERV expression, it is also possible for HERVs to get around this obstacle under conditions of cell stress or by the use of alternative transcription factors.

## 5. Examples of HERV Regulation

### 5.1. Embryonic and Induced Pluripotent Stem Cells

The regulation of HERVs is particularly critical during embryonic development and thus the expression of HERVs in stem cells is of great interest. There are two times when the genome undergoes an epigenetic ‘reset’ by the loss and subsequent re-establishment of DNA and histone methylation; the first is following fertilisation (partial reset, as some imprinted loci are protected from demethylation) and the second is during gametogenesis (full reset) [28]. These periods of global demethylation theoretically favour HERV activity since transcriptional repression is lost. Thus, a role for HERVs is likely during the embryonic stage as this is when there is de-repression of the proviral loci.

HERVs belonging to the HK2 and HERV-H families have been implicated in stem cell identity and embryonic development. Human embryonic carcinoma cells, such as the NCCIT cell line, have been used to model early development. In NCCIT cells, hypomethylation at a particular LTR belonging to the youngest HERV-K elements (LTR5HS), coupled with Oct4 binding, increased HERV-K expression [54]. The HERV-K ORFs were expressed and viral-like particles were produced [54]. In particular, the expression of the HERV accessory protein, Rec, modulates cellular mRNA expression and nuclear translocation [54]. Selective hypomethylation of HERV LTRs is therefore essential to regulate expression of HERVs required in early human development. In addition, HERV-H is associated with the active chromatin mark H3K4me3 in embryonic stem cells [55]. Further, the HERV-H LTR contains binding sites for the stem cell factor NANOG and sites for Oct4 and Sox2 are in close proximity [55]. Finally, HERV-H RNAs act as long non-coding RNAs (lncRNAs), which are important for the pluripotent identity of stem cells [56]. Thus, there is evidence of a role for the epigenetic regulation of HERVs in stem cells and embryonic development.

Given ethical considerations, it is difficult to study human embryonic development other than by using cell lines. It is thus not surprising that much more is known about the epigenetic regulation of ERVs during early developmental stages in mice. The KRAB-ZFPs are critical in this process, restoring methylation lost during fertilisation and thereby silencing ERV expression. ERVs are silenced early in embryonic development by the action of TRIM28/KAP1 [57]. The TRIM28 complex preserves imprinting marks and restores the methylation in the early mouse embryo [47]. In neural progenitor cells, TRIM28-mediated histone modifications repressed ERV expression; deletion of TRIM28 in these cells resulted in increased ERV expression as well as decreased H3K9me3 [58]. The knockdown of TRIM28/KAP1 also resulted in a loss of the repression and increased expression of ERVs in murine embryonic fibroblasts in an OCT4-GFP transgenic mouse model [59]. Similarly, in the production of induced pluripotent stem cells (iPSCs), TRIM28/KAP1 and SETDB1 act as barriers to reprogramming [59]. There is thus a critical role for the KRAB-ZFPs in suppressing ERVs which then leads to the loss of pluripotency.

### 5.2. Placenta and Pregnancy

It is well-established that HERVs contribute to formation of the placenta. The HERV-W env protein has been co-opted to serve as a fusion protein (called syncytin-1) critical to the formation of the syncytiotrophoblast [60]. Another co-opted *env* gene belonging to HERV-FRD encodes syncytin-2, which contributes to syncytiotrophoblast formation [61] and has a role in immune tolerance of the foetus [62]. A higher risk of pre-eclampsia was associated with reduced expression of both syncytin-1 and -2, with the reduction in syncytin-2 being more important [63]. Moreover, problems during gestational diabetes are linked to aberrant expression of syncytin-2 and its receptor, MFSD2 [64]. The role of syncytins in the placenta is discussed in detail in a recent review [65]; here, we are concerned with the epigenetic regulation of syncytin expression.

Both the HERV-W and HERV-FRD LTRs are controlled by histone H3 acetylation in placental tissues [66]. In addition, control of syncytin-1 expression is mediated by differential methylation. There is a global reduction in methylation levels in the placenta relative to other tissues, consistent with a high proportion of HERV LTRs acting as tissue-specific promoters in the placenta [67]. In particular, a CpG island in the 5′ LTR is hypomethylated in placental cells and hypermethylated in other tissues [68]. Over the course of a pregnancy, this CpG island becomes progressively more methylated [69]. Altered methylation of the HERV-W env locus and decreased expression of syncytin-1 have been observed in placentae from pre-eclampsia [61]. Exposure to oestrogens in the environment causes changes in the methylation of HERVs and this is linked to effects particularly on male children [70]. Aberrant expression of syncytin-1 in hydatiform moles has recently been described to contribute to malignant transformation [71]. Thus, altered epigenetic regulation of HERVs can lead to aberrant pregnancy and development.

There are lesser known roles for HERVs in fertility and pregnancy that merit further study. High levels of syncytin-2 are detected in the testes, which is another tissue that displays global hypomethylation and this is thought to favour HERV expression [65]. Syncytin-1 is also thought to be involved in fertilisation, possibly contributing to the fusion of gametes. Sperm express syncytin-1 on the cell surface whereas oocytes do not; instead, oocytes express the syncytin-1 receptor SLC1A5 [65]. Finally, HERV-K particles have also been detected in human placenta [72] but the functional significance of this, if any, remains unclear.

### 5.3. Cancer

There are numerous types of cancer and this makes it difficult to generalise about the contribution, if any, of HERVs to tumorigenesis. A number of papers do report a positive correlation between HERVs and cancers, while others find a lack of association [73]. Critically, the accessory proteins of HERV-K, Rec and Np9, have been associated with cancers [74,75,76] but also found to be expressed in normal tissues [77]. This illustrates some of the uncertainty in determining a causal relation between HERVs and cancer. A bystander effect might be useful in itself, permitting a HERV-based biomarker [78] or the use of HERV proteins as surrogate tumour antigens for therapeutic purposes [79,80]. A detailed analysis of the evidence for HERVs in cancer is beyond the scope of the current review; we will here limit ourselves to the contribution of epigenetic regulation. 

One common feature of cancer is a global hypomethylation of the genome; moreover, certain genes may be locally hypomethylated in cancer relative to normal tissues [81]. It is thus feasible that global and/or local hypomethylation leads to loss of repression of HERVs and there is evidence for this in a number of cancers. For example, there is a global hypomethylation of HERV-W and the LINE-1 retroelement in ovarian cancers [82] and hypomethylation of the HERV-K 5′ LTR is observed in melanomas [83]. Global hypomethylation does not necessarily correlate with expression of all HERVs. For example, the treatment of neuroblastoma cell lines with 5′-aza induced expression of multiple HERV-W loci [84], showing that a cancer cell line could still have HERVs that are suppressed by CpG methylation. Further, de-repression of HERV LTRs could lead to activation of otherwise silent oncogenes, a process referred to as ‘onco-exaptation’ [85]. Examples of such oncogenes induced following onco-exaptation of LTRs include tyrosine kinase receptors (ALK, ERBB4); in these examples, LTR-driven expression results in truncated proteins being produced and these are associated with cancers including lymphoma and melanoma [85].

Global hypomethylation could play a role in the expression of HERVs in GCT cell lines, such as the Tera-1 and NCCIT. These cell lines are known to produce HERV particles, with those of the NCCIT being mature particles that bud from the cells [86], while those from Tera-1 cells appear to lack the env glycoprotein [87]. In our lab, we found that the NCCIT cell line was particularly permissive to HK2 expression (manuscript in preparation), consistent with HERV expression and particle production by these cells. The NCCIT have a methylation pattern reminiscent of the pluripotent state [88] and, as they are an embryonic carcinoma cell line, they have been used to model early embryonic development [54]. While cancer cells and embryonic stem cells are clearly different, there is growing recognition of the common features of pluripotency and malignancy [26]. In particular, the global hypomethylation in tumour cells could be similar to that observed during early developmental stages. It is feasible that a stem cell-like phenotype is found in GCT [88], at least among the subset of cancer stem cells that are hypothesised to exist in most tumours to sustain cancer progression [89]. Since HERVs contribute to the identity of embryonic stem cells, they might also contribute to the formation of cancer stem cells. 

In addition, altered histone methylation or acetylation in cancer may contribute to de-repression of HERVs. This has been described in a recent analysis of repetitive elements in cancer cell lines using ENCODE ChIP-Seq data [16]. For example, increased HERV-Fc1 expression from a locus on chromosome 7 was found to be associated with active histone methylation [16]. One HERV LTR seems particularly sensitive to HDACi: the ERV9 LTR is present in thousands of copies in the human genome that is highly expressed in the male testes [90]. The expression of a testes-specific tumour suppressor protein, GTAp63, is under the regulation of the ERV9 LTR. This protein is absent in GCT but its expression can be induced in these cells by the use of the HDACi TSA and vorinostat [91]. Expression of GTAp63 induces apoptosis in these cells and is thought to be protective against GCT formation; the silencing of this protein contributes to tumour formation by preventing GTAp63-induced apoptosis [91]. This clearly indicates a role for histone acetylation in the control of GTAp63 expression and thus of ERV9 LTR promoter activity. In contrast, treatment with 5′-aza did not induce expression of GTAp63 and therefore CpG methylation is not involved in repression of this LTR. 

Interestingly, the ERV9 LTR was subsequently found to control the expression of several genes, many of which are involved in immunity or apoptosis [90]. The pro-apoptotic genes included TNFRSF10B, which encodes the death receptor 5 (DR5/Killer) protein [90]. The ERV9 LTR control of tumour suppressor genes in male germ cells reveals a protective effect of HERV LTR activity in preventing cancer. All of the ERV9 LTRs were activated by treatment with HDACi, leading the authors to propose the therapeutic use of HDACi to restore tumour suppression and induce apoptosis in GCT [90]. Of further note is the fact that HDACi did not increase the expression of other HERV subfamilies [90], suggesting that repression by histone deacetylation is not universal or, at least, not the sole mechanism of silencing. However, it is well-documented that HERVs are expressed in cell lines derived from GCTs [86] and we have measured HK2 expression in NCCITs. In contrast to Beyer and colleagues, we did detect a modest increase in HK2 expression with HDACi treatment (vorinostat, panobinostat) (manuscript in preparation). 

While HERVs are thought to have a contributory role in tumorigenesis, it is unlikely to be simply a matter of expression being on or off. The complexity of this is revealed by the analysis of genomic and transcriptomic data using new technologies. For example, the ENCODE ChIP-seq data revealed de-repression of certain repetitive elements including HERV-Fc1. However, of the seven loci of HERV-Fc1, only one was identified as having altered expression in cancer cell lines [16]. Moreover, the treatment of cells with inhibitors of such as 5′-aza or HDACi does not necessarily lead to increased HERV expression [30]. A fascinating twist in this story is that the use of DNA methylation inhibitors allows HERVs to be expressed and to trigger an innate immune response [92,93]. For example, the demethylation of HERVs leads to an immune response to dsRNA, producing exogenous interferon that could then prime neighbouring cells for immune checkpoint (anti-CTLA4) therapy [93]. Thus, the use of inhibitors of epigenetic modifications could prove beneficial in treating human cancers by harnessing HERV expression. 

## 6. Conclusions

Recent developments have revealed some of the complexities of HERV regulation by epigenetics. HERVs are not universally silenced; in normal physiology, there is a real need for HERV expression but this seems to be limited to particular tissues and times, with the key examples being placentation and embryonic development. As discussed, the expression of syncytin-1 contributes to the formation of the placenta and normal pregnancy. HERV-W, which encodes syncytin-1, has also been associated with neurological disorders and autoimmune diseases. This could be due to de-repression of the LTRs which could permit syncytin-1 expression in adult cells and this expression could be further enhanced by other stimuli. For example, cytokine-mediated transactivation of HERV expression has been described for HERV-W and HERV-K in ALS [20]. These data suggest a susceptibility of HERV LTRs to pro-inflammatory stimuli, allowing them to act in a positive way to amplify the immune response in the right context but possibly contributing to diseases such as ALS and multiple sclerosis (MS). A further example is the finding that HERV-W loci showed decreased association with H3K9me3 in the context of influenza virus expression, as well as transactivation by the transcription factor glial cells missing 1 (GCM1) [94]. It is thus feasible that exogenous virus infection could precipitate altered epigenetic marks and aberrant HERV expression in tissues where it is normally silenced. 

In addition, HERVs that are expressed may have a beneficial role in preventing cancer onset, such as by tumour suppression as in the case of the ERV9 LTR in male germ cells. Alternatively, it is still unclear to what extent HERVs may also contribute to tumour formation. For example, the expression of HERVs in GCT may be merely a consequence of the global hypomethylation but it is also possible that HERV expression somehow contributes to cancer onset or progression, such as through the action of the HERV-K accessory protein Rec. The altered epigenetic regulation in cancer cells may favour HERV expression, which could then have knock-on effects. The loss of epigenetic regulation at the level of the LTRs could allow the binding of transcription factors to consensus sites that are normally occluded. This has been described for the activation of oncogenes following onco-exaptation of HERV LTRs. These examples show that harnessing the potential of HERVs, particularly the HERV LTRs, comes at a potential cost should the epigenetic regulation be disrupted.
